# Editorial: Structure modification and activity evaluation of phytochemicals

**DOI:** 10.3389/fnut.2026.1805080

**Published:** 2026-02-24

**Authors:** Yongsheng Chen, Rian Yan, Yingbin Shen, Libo Tan

**Affiliations:** 1 Department of Food Science and Engineering, Jinan University, Guangzhou, China; 2 School of Life Sciences, Guangzhou University, Guangzhou, China; 3 Department of Human Nutrition, The University of Alabama, Tuscaloosa, AL, United States

**Keywords:** biological activities, functional food, nutrition, phytochemicals, structure activity relation, structure modification

Phytochemicals are a diverse and structurally complex group of bioactive compounds naturally present in plant-based foods, including fruits, vegetables, grains, and nuts. To date, several thousand dietary phytochemicals have been identified, and extensive evidence supports their important roles in human health, ranging from antioxidant and anti-inflammatory activities to metabolic, immune, and cellular protective functions (1, 2). Major classes of phytochemicals, such as carotenoids, polyphenols, isoprenoids, and phytosterols, have been widely studied for their biological activities and applications in functional foods and pharmaceutical development (3). Despite this progress, many phytochemicals remain underexplored, and their broader utilization is often constrained by challenges including low stability, poor solubility, and limited bioavailability.

Structural modification and processing optimization have therefore emerged as important strategies to enhance the functional properties of phytochemicals. By altering molecular structure through chemical derivatization or processing-induced modification, it is often possible to improve physicochemical properties while retaining or enhancing biological activity. Recent studies have demonstrated that targeted structural modification can significantly influence stability, bioavailability, and bioefficacy of natural compounds, indicating the importance of structure-activity relationship analysis in phytochemical research (4–6). Together, these approaches provide a foundation for advancing phytochemicals from promising bioactive molecules toward practical applications in nutrition, functional foods, and health-related interventions.

The Research Topic “*Structure Modification and Activity Evaluation of Phytochemicals*” was launched to highlight advances in this interdisciplinary field. The central objective of this Topic is to showcase how structural characterization, targeted modification, and functional evaluation can be integrated to better understand and enhance the biological properties of phytochemicals. The four articles included in this Topic collectively span analytical, experimental, and synthetic approaches, illustrating how structure-activity relationships can be investigated to improve the nutritional and health-related potential of plant-derived compounds.

One contribution focuses on the often-overlooked aerial parts of *Allium sativum*, which are typically discarded during harvest despite their rich phytochemical composition. Using a hyperlipidemic mouse model combined with advanced phytochemical profiling, the authors demonstrate that extracts from the aerial parts significantly improve lipid profiles and antioxidant status. This study highlights the importance of plant part selection and extraction strategies in identifying underutilized sources of bioactive phytochemicals, with potential implications for both nutritional applications and agricultural sustainability (Hu et al.).

Another original research article examines the impact of processing-induced structural modification on the biological activity of plant-derived polysaccharides. Using *Gastrodiae Rhizoma* as a model, the authors compare sulfur-fumigated and non-sulfur-fumigated materials and demonstrate that sulfur fumigation alters polysaccharide structure, leading to reduced antioxidant capacity. Through detailed structural and functional analyses, this work highlights how common processing techniques can unintentionally modify phytochemical structure and bioactivity, emphasizing the need to evaluate processing as a critical determinant of functional quality (Dai et al.).

Deliberate chemical modification is highlighted in a third contribution centered on luteolin, a widely distributed dietary flavonoid. By introducing acyl groups at specific hydroxyl positions, the authors generate a series of novel luteolin derivatives and systematically evaluate their biological activities. The modified compounds exhibit improved solubility and enhanced anti-inflammatory and antioxidant effects in experimental models, providing a clear example of how rational structural modification can improve the translational potential of phytochemicals while preserving their core bioactivity (Kong et al.).

Complementing these experimental studies, the Research Topic also includes a comprehensive systematic review of *Astragalus* species from the septentrional Algerian Sahara. This review synthesizes current knowledge on the phytochemical composition and reported biological activities of lesser-studied *Astragalus* species, with particular emphasis on saponins, phenolic compounds, and polysaccharides. By critically evaluating existing literature, the authors identify significant gaps in phytochemical characterization and functional validation, highlighting opportunities for future structure-focused and activity-driven research on traditionally used medicinal plants (Tedjani et al.).

Taken together, the articles in this Research Topic illustrate the diverse strategies available for advancing phytochemical research, from extraction optimization and processing evaluation to intentional chemical modification and systematic literature synthesis. A unifying theme across these contributions is the recognition that biological activity cannot be fully understood without careful consideration of chemical structure, whether modified through processing, derivatization, or natural variability among plant materials.

Looking ahead, continued progress in this field will depend on closer integration of analytical chemistry, synthetic modification, food science, and biologically relevant experimental models. Such interdisciplinary efforts are essential for translating phytochemicals from promising bioactive compounds into reliable components of functional foods, nutraceuticals, and therapeutic strategies (Figure 1). We hope this Research Topic will stimulate further research into structure-activity relationships and encourage the development of structure-informed approaches to maximize the health potential of phytochemicals.

**Figure 1:**
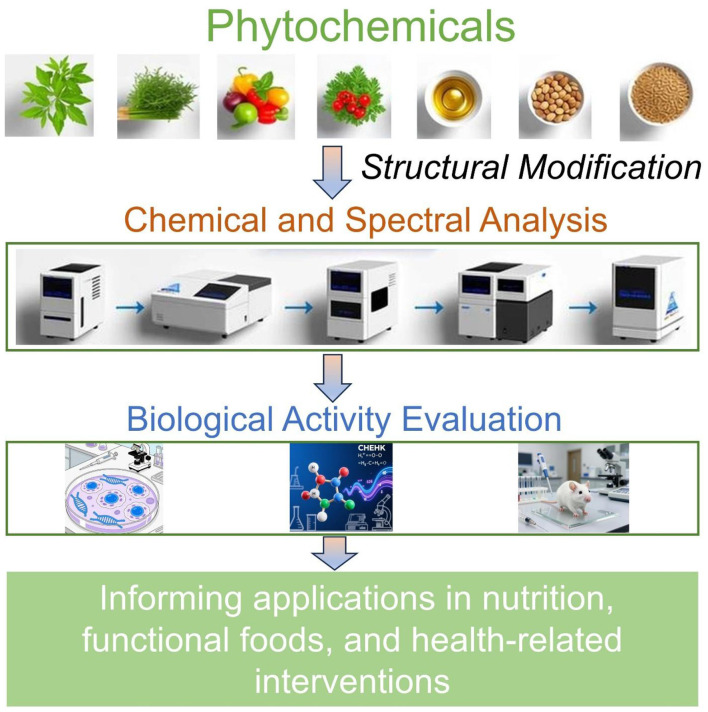
Conceptual framework illustrating the structure-activity-function pipeline in phytochemical research. Phytochemicals derived from plant and food sources may undergo structural modification through processing or targeted chemical derivatization. These structural changes are characterized by using analytical and spectroscopic approaches and evaluated for biological activity using *in vitro* and *in vivo* models, ultimately informing applications in nutrition, functional foods, and health-related interventions.

## References

[1] Pawase PA Goswami C Shams R Pandey VK Tripathi A Rustagi S . A conceptual review on classification, extraction, bioactive potential and role of phytochemicals in human health. Fut Foods. (2024) 9:100313. doi: 10.1016/j.fufo.2024.100313

[2] Pandohee J Kyereh E Kulshrestha S Xu BJ Mahomoodally MF. Review of the recent developments in metabolomics-based phytochemical research. Crit Rev Food Sci. (2023) 63:3734–49. doi: 10.1080/10408398.2021.1993127 34672234

[3] Chen YS Wang EP Wei ZH Zheng YF Yan R Ma . Phytochemical analysis, cellular antioxidant and α-glucosidase inhibitory activities of various herb plant organs. Ind Crop Prod. (2019) 141:111771. doi: 10.1016/j.indcrop.2019.111771

[4] Xu Q Deng H Li XT Quan ZS. Application of amino acids in the structural modification of natural products: a review. Front Chem. (2021) 9:650569. doi: 10.3389/fchem.2021.650569 33996749 PMC8118163

[5] Li H Chen Y Peng Q Tan X Chen G Zhou H . Flavonoids from bamboo leaves improve the stability of unsaturated fatty acids in the lipids of walnut emulsions. Ind Crop Prod. (2022) 178:114609. doi: 10.1016/j.indcrop.2022.114609

[6] Li HM Xu TT Peng QX Chen YS Zhou H Lu YY . Enzymatic acylation of rutin with benzoic acid ester and lipophilic, antiradical, and antiproliferative properties of the acylated derivatives. J Food Sci. (2021) 86:1714–25. doi: 10.1111/1750-3841.15703 33844282

